# Formation, preservation and extinction of high-pressure minerals in meteorites: temperature effects in shock metamorphism and shock classification

**DOI:** 10.1186/s40645-021-00463-2

**Published:** 2022-01-05

**Authors:** Jinping Hu, Thomas G. Sharp

**Affiliations:** 1 Division of Geological and Planetary Sciences, California Institute of Technology, Pasadena, CA 91125 USA; 2 School of Earth and Space Exploration, Arizona State University, Tempe, AZ 85287 USA

**Keywords:** Chondrite, Martian meteorite, Shock metamorphism, High-pressure minerals

## Abstract

The goal of classifying shock metamorphic features in meteorites is to estimate the corresponding shock pressure conditions. However, the temperature variability of shock metamorphism is equally important and can result in a diverse and heterogeneous set of shock features in samples with a common overall shock pressure. In particular, high-pressure (HP) minerals, which were previously used as a solid indicator of high shock pressure in meteorites, require complex pressure–temperature–time (*P–T–t*) histories to form and survive. First, parts of the sample must be heated to melting temperatures, at high pressure, to enable rapid formation of HP minerals before pressure release. Second, the HP minerals must be rapidly cooled to below a critical temperature, before the pressure returns to ambient conditions, to avoid retrograde transformation to their low-pressure polymorphs. These two constraints require the sample to contain large temperature heterogeneities, e.g. melt veins in a cooler groundmass, during shock. In this study, we calculated shock temperatures and possible *P–T* paths of chondritic and differentiated mafic–ultramafic rocks for various shock pressures. These *P–T* conditions and paths, combined with observations from shocked meteorites, are used to constrain shock conditions and *P–T*–*t* histories of HP-mineral bearing samples. The need for rapid thermal quench of HP phases requires a relatively low bulk-shock temperature and therefore moderate shock pressures below ~ 30 GPa, which matches the stabilities of these HP minerals. The low-temperature moderate-pressure host rock generally shows moderate shock-deformation features consistent with S4 and, less commonly, S5 shock stages. Shock pressures in excess of 50 GPa in meteorites result in melt breccias with high overall post-shock temperatures that anneal out HP-mineral signatures. The presence of ringwoodite, which is commonly considered an indicator of the S6 shock stage, is inconsistent with pressures in excess of 30 GPa and does not represent shock conditions different from S4 shock conditions. Indeed, ringwoodite and coexisting HP minerals should be considered as robust evidence for moderate shock pressures (S4) rather than extreme shock (S6) near whole-rock melting.

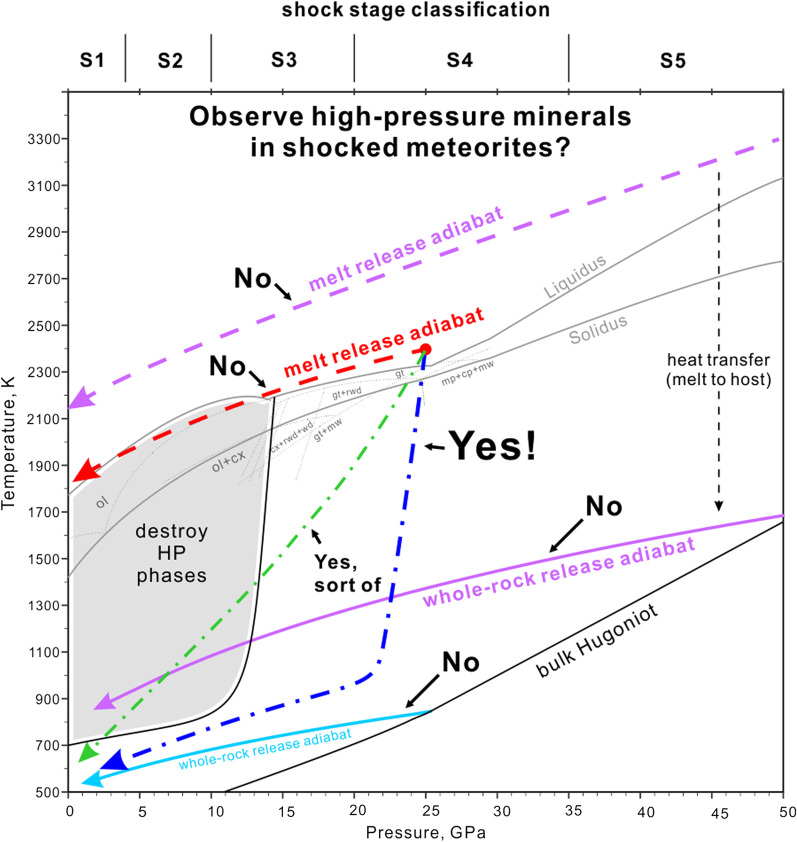

## Introduction

The study of shock metamorphism in natural samples is motivated by its usefulness for constraining planetary and terrestrial impact conditions, including the velocity, size and material of the impacting objects. High-pressure (HP) minerals have been recognized as unambiguous evidence of shock metamorphism in meteorites and terrestrial impact structures since the 1960s (e.g. Chao et al. 1960, 1962; Binns 1967; Binns et al. 1969). In ordinary chondrites and shergottites, for instance, the presence of HP minerals, combined with mineral recrystallization and the results of shock recovery experiments, was proposed as evidence for a very high shock stage (S6) near the whole-rock melting regime (e.g. Stöffler et al. 1986, 1991), which has been widely used by meteoriticists thereafter. In this paper, we will be discussing the controversy caused by this classification. Ringwoodite, the polymorph of olivine with spinel structure, is quite commonly found in the melt veins of highly shocked ordinary chondrites and Martian meteorites and occurs as deep blue grains that provide easily recognized evidence of high shock (Stöffler et al. 1991). A variety of HP minerals, including wadsleyite, majorite, akimotoite, bridgmanite, lingunite, etc. have provided natural examples of minerals thought to be present deep in Earth’s mantle (see Gillet and El Goresy 2013; Tomioka and Miyahara 2017 and references therein). Ahmed El Goresy has made a large contribution to the field of shock metamorphism and HP minerals through detailed petrographic and microanalytical studies of shocked samples and the discovery of new HP minerals (e.g. El Goresy and Chao 1976; Chen et al. 1996; El Goresy et al. 2004; Miyahara et al. 2019). Throughout his career, Professor El Goresy has been a strong proponent of using HP minerals to estimate shock pressures.

Although HP minerals provide clear evidence of high shock pressures in meteorites, their usefulness for constraining shock pressures is still debated because the pressures inferred from HP-mineral stabilities are far less than whole-rock melting and those inferred from deformational shock features and shock-recovery experiments (e.g., Stöffler et al. 1991; Sharp and De Carli 2006; Gillet and El Goresy 2013; Fritz et al. 2017).

HP minerals in shocked meteorites form by the crystallization of shock melt and by solid-state transformation of mineral fragments entrained in or in contact with shock melt (e.g. Binns et al. 1969; Chen et al 1996). Chen et al. (1996) were the first to recognize that the melt-vein crystallization assemblage, in the L6 chondrite Sixiangkou, matched the liquidus assemblage in the high-pressure melting experiments on Allende (Agee et al., 1995) and peridotite (Zhang and Herzberg, 1994). They concluded that the melt veins in Sixiangkou crystallized at 20–24 GPa and that this represented the equilibrium shock pressure for the sample. Since then, many have used shock-melt crystallization assemblages to constrain shock pressure or pressure during decompression (e.g. Sharp et al. 1997; Langenhorst and Poirier 2000; Beck et al. 2004; Ohtani et al. 2004; Xie and Sharp 2004; Xie et al 2006a, 2006b; Chen and Xie 2008; Fritz and Greshake 2009; Miyahara et al. 2011a; Walton et al. 2014; Hu and Sharp 2017; Pang et al. 2016; Li and Hsu 2018 and many more), but this approach has not been universally adopted because of questions regarding the use of equilibrium phase diagrams to interpret shock pressure. Although supercooling and metastable crystallization can occur within the shock melt (Hu and Sharp 2017), the crystallization assemblages in most chondritic samples are consistent with the liquidus assemblages produced in static experiments (Zhang and Herzberg 1994; Agee et al. 1995; Herzberg and Zhang 1996; Trønnes and Frost 2002). If the shock melt remains in a liquid state after pressure release, it crystallizes a low-pressure assemblage (Xie et al. 2006b), such as olivine, orthopyroxene plus plagioclase, mostly observed in for example martian meteorites, which generally show evidence of shock pulses (~ 10 ms) that are 1–2 orders of magnitude shorter than those inferred for shocked L chondrites (Langenhorst and Poirier 2000; Ohtani et al. 2004; Beck et al. 2005; Walton et al. 2014; Sharp et al. 2015;). Solid-state transformations of olivine and pyroxene only occur in association with shock melt and are strongly dependent on large temperature heterogeneities. Because of the short duration of shock-induced high-pressure pulses (milliseconds to seconds) in hypervelocity impacts, particularly for Martian meteorites (e.g. Beck et al. 2005; Walton et al. 2014; Sharp et al. 2015, 2019), the reconstructive phase transformations of the mineral constituents to their HP polymorphs must occur very quickly. Transformation temperatures close to melting are required to achieve the necessary reaction rates during the shock pulse (Xie and Sharp 2007). This explains the nearly exclusive association of HP minerals with shock-melt veins and pockets in meteorites.

Many other features are recognized as indicators of shock in meteorites, including planar fractures, planar deformation features, mosaicism, staining and recrystallization of olivine (e.g. Bauer 1979; Dodd and Jarosewich 1979; Stöffler et al. 1991; Takenouchi et al. 2019) plus blackening of silicates by disseminated metal or sulfide (e.g. Heymann 1967; Rubin, 1992; Moreau et al. 2017), maskelynite and feldspathic normal glass (e.g. Milton and DeCarli 1963; Stöffler 1984; Ferrière and Brandstätter 2015) and pervasive shock melt (e.g. Fredriksson et al. 1963; Dodd and Jarosewich 1979). The shock classification systems of Stöffler et al. (1991, 2018) use progressive shock effects to classify shocked meteorites as shock stages S1 (unshocked, < 5 GPa) through S6 (very highly shocked, < 75 GPa; the pressure thresholds vary with rock type) and whole-rock melting. The shock pressures needed to produce these shock effects are calibrated against shock effects observed from shock-recovery experiments (e.g. Milton and DeCarli 1963; Stöffler 1972; Kieffer et al. 1976; Bauer 1979; Jeanloz 1980; Ostertag 1983; Stöffler and Langenhorst 1994).

A variety of shock features, including shock melt, commonly co-exist in the so-called impact melt breccias and strongly shocked martian meteorites, which are rich in quenched impact melt and contain lithic and mineral clasts of the impacted rock. However, HP minerals are consistently absent in these melt-rich breccia and achondrite samples. This suggests that either the pressures in these samples were not sufficiently high to generate HP minerals, or the post-shock temperature was too high for the preservation of metastable high-pressure minerals (Stöffler 1974). The former is unlikely because the impact breccias (Hu 2016) and strongly shocked shergottites (Walton and Herd 2007) generally contain more pervasive mosaicism, extensive feldspar glass, silicate blackening and more melting than HP-mineral bearing S6 samples. It is more likely that the shock features and high-pressure minerals are annealed by high post-shock temperatures during slow cooling.

Unlike olivine recrystallization that can be unambiguously recovered from experimental shock at high pressure (e.g. Bauer 1979), HP minerals were first proposed and used as indicator of S6 extreme shock (Stöffler et al. 1991) because of the excessive difficulty in forming them by shock-synthesis (Stöffler 1974; Syono et al. 1981; Tschauner et al. 2009). More recently, HP minerals were excluded from a revised shock-stage scheme (Stöffler et al. 2018) because their complex formation conditions require specific *P–T*–*t* paths instead of just certain shock pressure (Fritz et al. 2017). This leaves out an important source of information about shock conditions. If shock pressure alone cannot describe the formation of HP minerals, it is worth reviewing exactly what shock conditions they can constrain in a multi-parameter space. High pressures and temperatures in the shock state are necessary, but not fully sufficient, to produce observable HP minerals in shocked meteorites. Cooling of the meteorite before or during pressure release is equally important for the preservation of HP phases (e.g. Walton 2013; Hu and Sharp 2017; Sharp et al. 2019; Hu et al. 2020). The HP phases formed during the initial shock pulse are metastable at low pressure and they will be transformed back to low-density phases if the temperature is sufficiently high for retrograde back transformation after pressure release. HP minerals are only preserved if the sample cools through a critical temperature before the pressure release makes them metastable. That critical temperature is dependent on the thermodynamic stability of the mineral and on the reaction rates for back transformation (Reynard et al. 1996; Kimura et al. 2003; Hu and Sharp 2017), which sets an upper bound for the post-shock temperature of the sample. In summary, the HP phases observed in meteorites require a pressure–temperature–time (*P*–*T*–*t*) path that involves very high shock temperatures to drive high-pressure reactions, combined with rapid thermal quench during decompression to prevent back-transformation reactions at low-pressure. An understanding of *P–T*–*t* histories of HP-mineral bearing samples provide an opportunity to further constrain shock conditions in highly shocked meteorites if shock temperatures can be accurately estimated.

Overall, natural shock features, particularly HP minerals, in meteorites result from shock-induced high pressures plus complex and heterogeneous thermal histories. In this study, we calculated the bulk shock temperatures and release paths of common rocks and meteorites and use these to re-evaluate the shock conditions and *P*–*T*–*t* paths in highly shocked chondrites and achondrites in the context of progressive shock-stage classification. We are mostly focused on the temperatures of shock metamorphism, the formation condition of HP minerals and the *P–T*–*t* paths necessary for their preservation. For reviews on shock physics of geological materials, impact processes, HP mineral physics, shock stage classifications and summary of HP minerals in meteorites, the readers are referred to Melosh (1989), Stöffler et al. (1991), Sharp and DeCarli (2006), Gillet and El Goresy (2013), Asimow (2015), Fritz et al. (2017), Tomioka and Miyahara (2017) and Stöffler et al. (2018).

## Calculation of shock temperatures

Since the 1950s, shock experiments have been used to obtain high-pressure equation of state (EOS) data for various materials. Commonly, those experiments measure the velocity histories of the sample material in the shock state (e.g. Goranson et al. 1955; Walsh and Christian 1955; Jones et al. 1966). The corresponding pressure and density of the shocked material can be precisely calculated with the Rankine–Hugoniot relations for a simple one-wave shock. However, the measurement of shock temperatures is more complicated and the available techniques only work for limited types of samples (e.g. Asimow 2018 and references therein). Moreover, the calculation of shock temperatures requires high order equations of state with considerable assumptions and approximations. In order to discuss the temperature effects in naturally shocked meteorites, we introduce two methods for calculating shock temperatures. The first method employs the Birch-Murnaghan and Mie-Grüneisen equation of state. The second method uses an integral approximation along the Hugoniot of the sample.

### Shock temperature in EOS

Assuming the pre-shock velocity of the sample is zero, from conservation of mass, momentum and energy in shock state, the Rankine–Hugoniot equations can be written as:

1 $$\rho_{{\text{o}}} U_{{\text{s}}} = \rho \left( {U_{{\text{s}}} - u_{{\text{p}}} } \right)$$

2 $$\left( {P - P_{{\text{o}}} } \right) = \rho_{{\text{o}}} U_{{\text{s}}} u_{{\text{p}}}$$

3 $$E - E_{{\text{o}}} = \left( {P + P_{{\text{o}}} } \right)\left( {V - V_{{\text{o}}} } \right)/2$$

where *ρ* is the density, *U*_s_ is the shock-wave velocity, *u*_p_ is the particle velocity, *P* is the pressure, *V* is the specific volume (inverse density *ρ*) and *E* is the internal energy per specific volume. The subscript 0 denotes the pre-shock state of the sample. The Rankine–Hugoniot equations work for time-invariant systems. In the case that the sample has an initial velocity of *u*, *u*_p_ in Eqs. (1)–(3) needs to be replaced by *u*_p_ − *u*. Correlations between the state functions *P*, *V*, *E*, *u*_p_ and *U*_s_ in the shock state are called the Hugoniot curve of the material, or simply the Hugoniot.

**Fig. 1:**
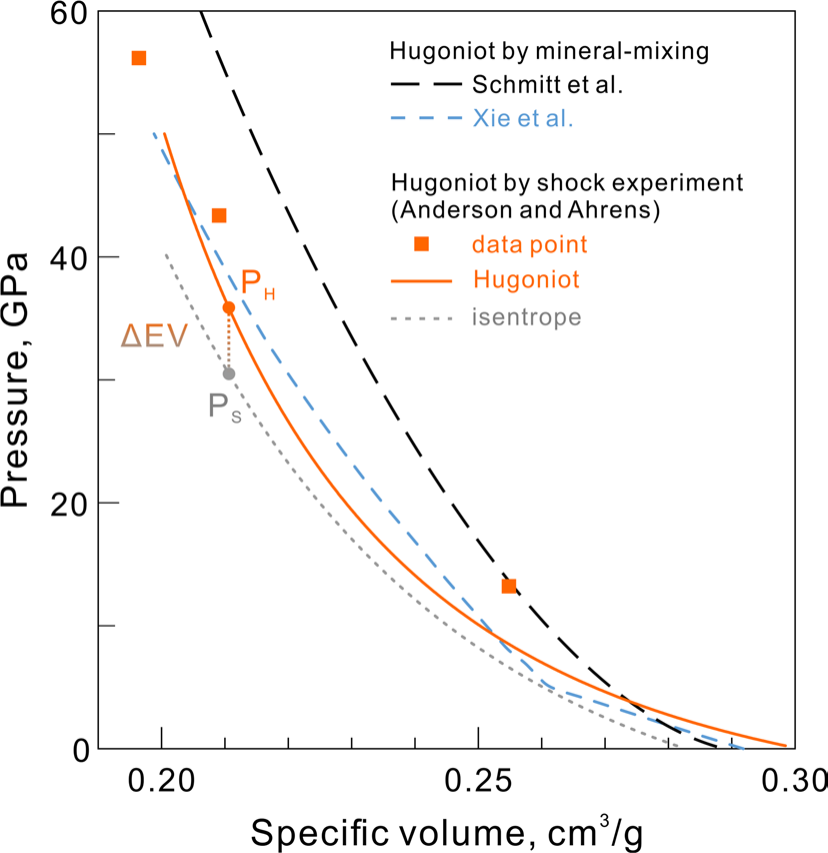
Pressure–volume Hugoniots of L chondrite from mineral mixing models (Schmitt et al. 1994; Xie et al. 2006b) and shock experiment (Anderson and Ahrens 1998) along with a calculated dense isentrope. Δ*E*_V_ is the internal energy difference between the isentropic and Hugoniot state of the same specific volume

For a plastic wave propagating in a single-phase solid material, *U*_s_ and *u*_p_ yield an empirical linear relationship:

4 $$U_{{\text{s}}} = C_{{\text{o}}} + s\,u_{{\text{p}}}$$

where *C*_o_ is the zero-pressure sound speed of the material and *s* is a dimensionless factor. This form of Hugoniot is convenient because *C*_o_ and *s* are obtained by fitting the velocity data measured in shock experiments (Table 1). For demonstrating the shock behavior of material, it is more straightforward to show the pressure–volume correlation (Fig. 1) calculated by Eq. (1), (2) and (4).

**Table 1:** Hugoniot data for basalt, gabbro and chondrites

	Kinosaki basalt^1^	Murchison^2^ CM2	Bruderheim^2^ L6	San Macros Gabbro^3^
*ρ*_o_, kg/m^3^	2700	2244	3337	2941
porosity, vol%	4–7%	23%	6–8%	–
*K*_oS_, GPa	50	24	32	32
*K*_oS_'	4.2	4.9	5.5	4.6
*γ*_o_	1.39	1	2.00	1.37
*q*	–	0	–	0.94
*C*_o_, km/s	3.5	1.87	3.11	3.3
*s*	1.3	1.48	1.62	1.41

*ρ*_o_: initial bulk density, *K*_oS_: zero-pressure isentropic bulk modulus, *K*_oS_': pressure derivative of the bulk modulus, *γ*_o_ and *q*: Grüneisen parameter at initial crystal density and the exponent factor, *C*_o_ and *s*: bulk sound speed and the dimensionless parameter in the *U*_S_-*u*_P_ Hugoniot

^1^Sekine et al. (2008); ^2^Anderson and Ahrens (1998); ^3^Boslough (1984)

If the sample undergoes a phase transformation in the shock state, separate Hugoniot curves for the low-density and high-density phases are needed to describe the shock behavior. Porosity also complicates the shock behavior and Hugoniot of a material. In the low-pressure regime, pores are collapsed by an infinitely weak shock, leading to a gentle slope on the P–V Hugoniot (Fig. 1). In practice, it is not uncommon for polymineralic porous rocks to have nearly linear *U*_s_-*u*_p_ Hugoniot (Table 1) in a wide range of pressures above the Hugoniot elastic limit (e.g. Ahrens and Gregson 1964; Anderson and Kanamori 1968; Marsh 1980; Anderson and Ahrens 1998; Sekine et al. 2008).

Although shock wave propagation can be considered as adiabatic at large spatial scales, the discontinuity between pre-shock and shock states increases the entropy of the system. Derivation of the Rankine–Hugoniot equations show that the entropy increase along the Hugoniot is of third order (McQueen 1989). In other words, the first- and second-order pressure/volume derivative on the isentrope and Hugoniot are the same at zero pressure. Therefore, at low pressure, the Hugoniot of a non-porous material and its isentrope are close to each other (Jeanloz 1989; McQueen 1989). To obtain the temperature on the Hugoniot, it is useful to first calculate the temperature on the isentrope and make a correction towards the Hugoniot.

To demonstrate the pressure–volume correlation of isentropic compression, the third order Birch-Murnaghan finite-strain equation of state is widely used (Jeanloz 1989) in its isentropic form:

5 $$P_{{\text{S}}} = 3K_{{{\text{oS}}}} f\left( {1 + 2f} \right)^{\frac{5}{2}} \left( {1 + \frac{3}{2}\left( {K_{{{\text{oS}}}}^{^{\prime}} - 4} \right) f} \right)$$

*P*_S_ is the pressure on the principle isentrope, *K*_oS_ is the zero-pressure isentropic bulk modulus, *K*_oS_′ is the pressure derivative of *K*_oS_ and

6 $$f = \frac{1}{2}\left[ {\left( {\frac{{V_{{\text{o}}} }}{V}} \right)^{\frac{2}{3}} - 1} \right]$$

For porous samples, the *V*_o_ and *K*_oS_ need to be the zero-pressure specific volume and bulk modulus of the material in the condensed state. With the limit of zero strain (Ruoff 1967), *K*_oS_ and *K*’_oS_ are obtain by

7 $$K_{{{\text{oS}}}} = C_{{\text{o}}}^{2} \rho_{{\text{o}}}$$

8 $$K_{{{\text{oS}}}}^{^{\prime}} = 4s{-}1$$

although with high finite strain, the effect of *K*_oS_″ becomes significant and causes the *K*_oS_′ to deviate from 4*s* − 1 by 20% (Jeanloz 1989).

Since our goal is to determine the temperature, the total differential of entropy is written as a function of *V* and *T* and combined with the *Maxwell’s relation*:

9 $${\text{d}}S\left( {V,T} \right) = \left( {\frac{\partial S}{{\partial T}}} \right)_{V} {\text{d}}T + \left( {\frac{\partial P}{{\partial T}}} \right)_{V} {\text{d}}V$$

On the isentrope, d*S* = 0 leads to

10 $$\left( {\frac{\partial S}{{\partial P}}} \right)_{V} {\text{d}}T = - {\text{d}}V$$

Temperature and volume are two independent variables in Eq. (10). However, the solution for the differential d*T* and d*V* is difficult because pressure and entropy are two parameter (*V*, *T*) variables.

To solve the differential equation, we use the well-known Grüneisen parameter (*γ*). It is a parameter to describe the thermal pressure at constant volume, by its definition:

11 $$\gamma = V\left( {\frac{\partial P}{{\partial E}}} \right)_{V}$$

The Grüneisen parameter has the advantage of being well-approximated as a function of volume only and can be used to relate other thermodynamic parameters, e.g. *S* and *P* in Eq. (10). The *γ* *−* *V* correlation is commonly expressed as:

12 $$\gamma \left( V \right) = \gamma_{o} \left( {\frac{V}{{V_{o} }}} \right)^{q}$$

where *γ*_o_ is the initial Grüneisen parameter at volume *V*_o_ and *q* is an exponential factor. Applying the first law of thermodynamics, d*E* = *T*d*S *− *P*d*V*, to Eq. (11), we get

13 $$\left( {\frac{\partial P}{{\partial S}}} \right)_{V} = \frac{\gamma T}{V}$$

The combination of Eqs. (10), (12) and (13) demonstrates that the temperature (*T*_S_) on the isentrope at a given *V* is solved as

14 $$T_{S} = T_{o} e^{{\frac{{\gamma_{o} }}{q}\left[ {1 - \left( {\frac{V}{{V_{o} }}} \right)^{q} } \right]}}$$

and *T*_o_ is the starting temperature.

Knowing the isentropic pressure (*P*_S_) and temperature (*T*_S_) at a certain volume V, correction to the Hugoniot pressure (*P*_H_) and temperature (*T*_H_) at this given volume V can be done by solving the Grüneisen Eq. (11). We get:

15 $$\Delta E_{{\text{V}}} \left( V \right) = E_{{\text{H}}} \left( V \right) - E_{{\text{S}}} \left( V \right) = \frac{V}{\gamma \left( V \right)}\left( {P_{{\text{H}}} \left( V \right) - P_{{\text{S}}} \left( V \right)} \right)$$

where Δ*E*v is the energy difference (Fig. 1) between the system energy on the Hugoniot (*E*_H_) and isentrope (*E*_S_). This is referred to as the Mie-Grüneisen equation of state. Thus, the temperature on the Hugoniot is given by

16 $$T_{{\text{H}}} = T_{{\text{S}}} + {\Delta {T_{{\text{V}}}}} = T_{{\text{S}}} + \frac{{\Delta E_{{\text{V}}} \left( V \right)}}{{C_{{\text{V}}} }}$$

where Δ*T*v is the conceptional temperature difference between the Hugoniot and isentrope at a given volume and *C*_V_ is the isochoric heat capacity defined by the temperature partial derivative of internal energy at constant volume.

**Fig. 2:**
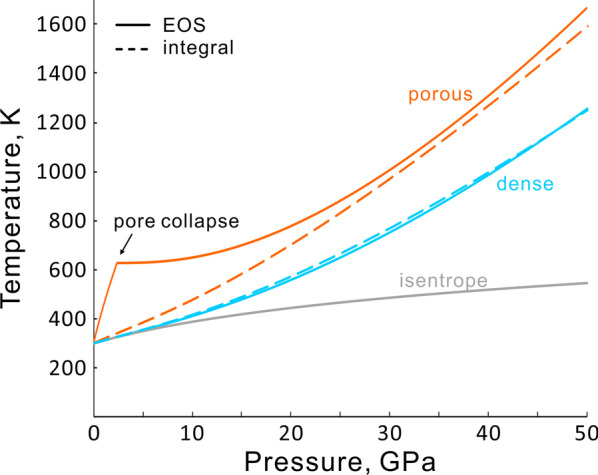
The shock temperature versus pressure for porous and dense L chondrite, calculated by the equation of state (EOS) and integral approximation, using the Hugoniot data in Table 1

As mentioned, *T*_S_ and *T*_H_ are close for condensed material at below 10 GPa (Fig. 2). The effect of porosity in the sample will be discussed in Sect. 2.3.

### Shock temperature by integral approximation along the Hugoniot

The application of the Birch-Murnaghan EOS requires the ability to fit the shock velocity data to the condensed isentropic bulk modulus. For a polymineralic rock of heterogeneous textures, this fitting can have considerable uncertainty if the experimental data is too limited (Fig. 1). An alternative method for estimating the shock temperature is to integrate the infinitesimal temperature change along the Hugoniot to the pressure of interest (McQueen 1989). The advantage of this method is that it makes use of the measured Hugoniot curve and does not required fitting of the data to linear *U*_S_-*u*_p_ correlations.

From the Hugoniot energy Eq. (3) and the first law of thermodynamics, the total differential of internal energy on the Hugoniot is

17 $${\text{d}}E = \frac{1}{2}\left[ {\left( {V_{o} - V} \right){\text{d}}P - P{\text{d}}V} \right] = T{\text{d}}S - P{\text{d}}V$$

Using the isochoric heat capacity and Grüneisen parameter to replace the partial derivatives in Eq. (9), the differential of entropy is written as

18 $${\text{d}}S\left( {V,T} \right) = \frac{{C_{V} }}{T}{\text{d}}T + \frac{{\gamma C_{V} }}{V}{\text{d}}V$$

Substituting the d*S* in Eq. (17), we get

19 $${\text{d}}T = - T\frac{\gamma }{V}{\text{d}}V + \frac{1}{{2C_{{\text{V}}} }}\left[ {\left( {V_{{\text{o}}} - V} \right){\text{d}}P + P{\text{d}}V} \right]$$

A general solution for *T* in this differential equation is mathematically difficult. However, in practice, we can replace the infinitesimal d*V* and d*P* by a small Δ*V* and Δ*P* on the Hugoniot (McQueen 1989). Assuming *i* and *i* − 1 are two closely adjacent points on the Hugoniot, Eq. (19) is approximated by

20 $$T_{i} = \frac{{T_{i - 1} \left[ {1 - \frac{{\overline{\gamma }\left( {V_{i} - V_{i - 1} } \right)}}{{2\overline{V}}}} \right] + \frac{1}{{2\overline{{C_{{\text{V}}} }} }}\left[ {\left( {V_{o} - \overline{V}} \right)\left( {P_{i} - P_{i - 1} } \right) + \overline{P}\left( {V_{i} - V_{i - 1} } \right)} \right]}}{{1 + \frac{{\overline{\gamma }\left( {V_{i} - V_{i - 1} } \right)}}{{2\overline{V}}}}}$$

where the overbar denotes the mathematical average of *V*, *P*, *γ* and *C*_V_ between points *i* and *i* − 1.

Unlike the B–M equation of state, the integral approximation is not a function of the final state on the Hugoniot. Every shock temperature on the Hugoniot is important for calculating the temperature of the next points. For condensed material, the results from the two methods generally agree well (Fig. 2) and both give reasonable estimates of shock temperature.

### Shock temperature and porosity of chondritic and differentiated mafic rocks

The Hugoniot of common rocks and meteorites used for temperature calculation is summarized in Table 1. The *U*_s_-*u*_p_ data of the Bruderheim L6 chondrite does not perfectly fit a single line (Anderson and Ahrens 1998). At 25–65 GPa, the Hugoniot seems to be a mixture of low-density and high-density phases. Also, at ~ 13 GPa the shock wave shows a two-wave structure, commonly interpreted as a low velocity shock wave overlapping with an elastic precursor. However, the velocity is extraordinarily high for an elastic precursor of silicate rock and, alternatively, may result from a sluggish phase transformation (Fig. 1; Anderson and Ahrens 1998). The data were not enough to fit separate Hugoniots for the phase transitions. Nevertheless, the low- and high-density Hugoniots are not drastically different, probably because either one is a combination of low- and high-density polymorphs of rock-forming minerals. The simplified fitted Hugoniot generally matches the data points except at low-pressure < 13 GPa (Fig. 1). Given that, and the very limited Hugoniot data for L chondrites, we still use the linear Hugoniot that reasonably fits the HP data points for our calculation. Hugoniot of terrestrial mafic rocks, such as dunite, peridotite and gabbro do not seem to resemble the chondrite data in phase transition (Marsh 1980). Mineral-mixing models of chondrite can also be used to fit and interpret the Hugoniot data. A mineral-mixing model by Schmitt et al. (1994) shows a Hugoniot above the measurements. In contrast, the mixing model for Tenham L6 chondrite used by Xie et al. (2006b) and Sharp and DeCarli (2006) agrees with the fitted Hugoniot (Fig. 1). This suggests that the fitted Hugoniot and the model by Xie et al. (2006b) are likely suitable for temperature calculations.

The Grüneisen parameter of rocks is an essential part in temperature calculations. The zero-pressure *γ*_o_ can be calculated by

21 $$\gamma = \frac{{V\alpha K_{{\text{T}}} }}{{C_{{\text{V}}} }}$$

where *α* is thermal expansion and *K*_T_ is the isothermal bulk modulus (Asimow 2015). The high-pressure *γ* and exponential factor *q* can be determined by shocking material of different initial conditions to the same volume (e.g. Luo et al. 2002). If experimental data is not available, it is reasonable to assume *q* = 1, thus *ργ* = *ρ*_o_*γ*_o_, for solids (Asimow 2012).

The isochoric heat capacity *C*_V_ is also needed for the calculations (Eq. 16 and 20). In experiments, the isobaric heat capacity *C*_P_ is commonly measured. They have the correlation of

22 $$C_{{\text{P}}} - C_{{\text{V}}} = TV\alpha^{2} K_{{\text{T}}}$$

For typical solids, the difference between *C*_V_ and *C*_P_ are on the order of 10 J/kg K (Navrotsky 1994). The pressure effect on heat capacity has not been fully investigated. Data on olivine suggest that at the pressure of interest the heat capacity decreases on the order of 10 J/kg K compared to that of zero pressure. The measured *C*_P_ values at low pressure are in the range of 800–1400 J/kg K at 300–3000 K for common rocks and silicate minerals (Watanabe 1982; Richet and Fiquet 1991; Waples and Waples 2004). We use these values for our calculations although we understand that they are somewhat different from the needed high-pressure *C*_V_.

Natural samples, other than deep intrusive rocks, have considerable initial porosity (Table 1). A porous Hugoniot can be calculated from the Hugoniot of a condensed material using the following equation (e.gAhrens and O’Keefe 1972; Tyburczy et al. 2001):

23 $$P_{{\text{p}}} = P_{{\text{d}}} \frac{{1 - \frac{\gamma }{2}\left( {\frac{{V_{{{\text{od}}}} }}{V} - 1} \right)}}{{1 + \frac{\gamma }{2}\left( {\frac{{V_{{{\text{op}}}} }}{V} - 1} \right)}}$$

where *P*_p_ and *P*_d_ are the shock pressure on porous and dense Hugoniot at volume V. *V*_od_ and *V*_op_ are the initial volume of dense and porous sample. We also use this equation and the Hugoniot of naturally porous samples to estimate the zero-pressure bulk modulus of the same material in the condensed state (Table 1).

Figure 1 shows the Hugoniot of a naturally porous L chondrite plus the calculated (dense) reference isentrope. The dense isentrope matches with the pore collapse volume predicted by the mineral-mixing model from Xie et al. (2006b). The corresponding shock temperatures are included in Fig. 2, calculated by the two methods introduced in Sects. 2.1 and 2.2. The temperature estimates of dense material from the two methods are close to each other. The small difference likely suggests the uncertainty of bulk modulus calculated from the simplified Hugoniot.

In contrast, the EOS calculation for a porous Hugoniot shows a temperature jump in the pore collapse regime. This is because the pore collapse by the infinite weak shock deposits large amount of energy to the system and increases the shock temperature. In the Mie-Grüneisen EOS, this process is quantitatively demonstrated by the fact that the Hugoniot pressure and isentropic pressure (infinite weak) at a given volume are significantly different for porous material (Fig. 1). Thus, the Δ*E*_V_ and *T*_V_ in Eqs. (15) and (16) are greater than that of condensed material. The integral approximation does not show such a temperature jump because this method relies on the shape of the Hugoniot curve. The simplified Hugoniot of L chondrite (Table 1) does not include a pore-collapse transition, which is thus neglected in the temperature calculation. In the integral method, each temperature is dependent on the temperature of the adjacent point. Therefore, the underestimation of shock temperature in the pore collapse regime is propagated into the high-pressure temperatures. This explains why the integral temperatures are below the EOS temperatures for the porous material. Because there is not enough data to constrain the shock behavior of a chondrite through the pore-collapse and the elastic regimes, neither of the two methods is perfectly accurate. Nevertheless, both methods provide reasonably consistent shock temperature approximations of porous materials.

**Fig. 3:**
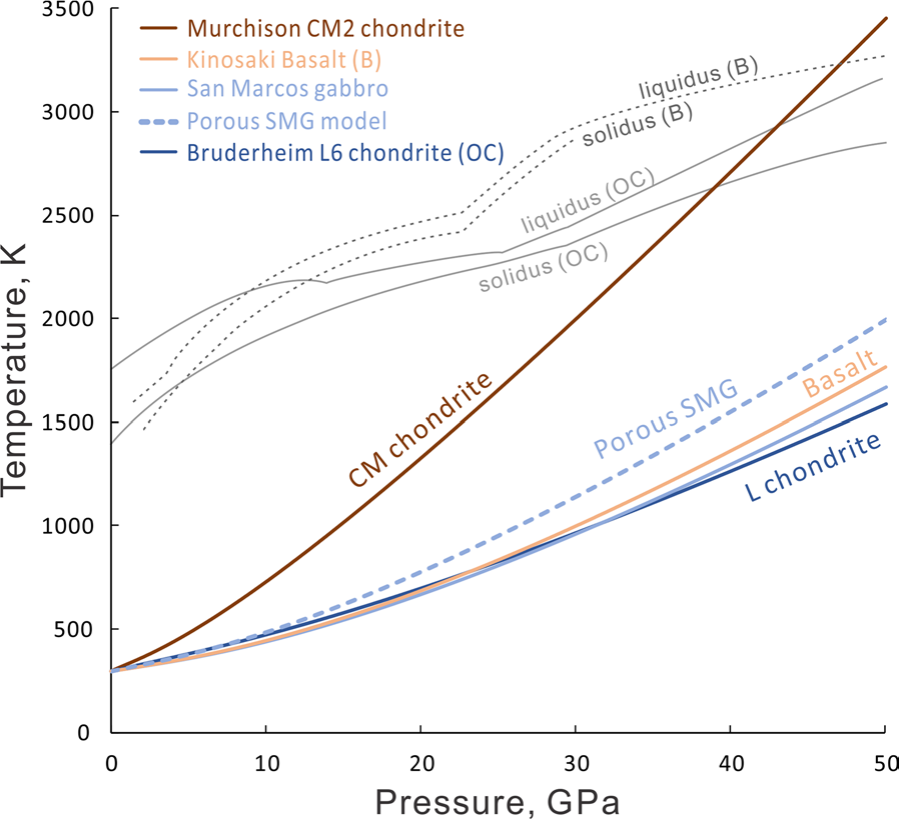
The shock temperatures of common rocks and meteorites, calculated by the Hugoniot integral approximation, using the parameters in Table 1. SMG is San Marcos gabbro. The representative solidus and liquidus of ultramafic/mafic rocks are for chondrites (OC) from Agee et al. (1995) and basalt (B) from Hirose et al. (1999) and Andrault et al. (2011)

Shock temperatures for common rocks calculated by the integral method, and all starting with 298 K, are shown in Fig. 3. The Murchison CM2 chondrite has a distinctly higher porosity (Table 1) and therefore higher shock temperature. The *P*–*T* Hugoniot crosses the chondrite liquidus at 40 GPa. For other rock types, whole-rock melting does not occur below 50 GPa, but melting may still occur locally, forming melt veins and pockets. The shock temperatures roughly increase in the sequence of L chondrite, dense gabbro, basalt and porous gabbro. This sequence agrees with increasing porosity and compressibility of these rocks. These results are consistent with results using similar calculation methods by Gillet and El Goresy (2013).

## Temperatures on release adiabat

Post-shock temperature can have a significant effect on annealing high-pressure signatures in shocked meteorites (Rubin 2002, 2004; Kimura et al. 2003; Walton 2013; Hu and Sharp 2017; Fukimoto et al. 2020). Annealing used in the context of shock metamorphism refers to the retrograde metamorphism of shock features resulting from post-shock heating (Bauer 1979; Jeanloz 1980). The post-shock temperature for a given shock pressure can be calculated from the amount of waste heat added to the system, defined by the work from shock loading along Rayleigh-line minus the work from decompression (Schmitt et al. 1994; Sharp and DeCarli 2006). However, the final post-shock temperature is not the only important factor in the retrograde metamorphism of shock features. On the release path, a high-*T* moderate-*P* condition may also modify the signature of peak shock pressure (Hu and Sharp 2017). In that case, it is useful to calculate the *P*_release_-*T*_release_ path of continuous decompression.

As the release wave propagates, decompression occurs as a continuous process in a time-variant system (Asimow 2015). Several techniques are used to measure the release states in shock experiments, e.g. using a series of release buffers that have lower shock impedance than the sample to get a series of *u*_p_-*P* points on the release path (Anderson and Ahrens 1998; Luo et al. 2003). The volume on the release path can be calculated with the Riemann integral

24 $$V_{{\text{r}}} = V_{{\text{H}}} + \mathop \smallint \limits_{{u_{{{\text{pH}}}} }}^{{u_{{{\text{pr}}}} }} \frac{{{\text{d}}u_{{\text{p}}} }}{{{\raise0.7ex\hbox{${{\text{d}}P}$} \!\mathord{\left/ {\vphantom {{{\text{d}}P} {{\text{d}}u_{{\text{p}}} }}}\right.\kern-\nulldelimiterspace} \!\lower0.7ex\hbox{${{\text{d}}u_{{\text{p}}} }$}}}}$$

where H and r denotes the points on the Hugoniot and release path. The release adiabat is approximately isentropic so that the temperature upon decompression can be calculated with a modification of Eq. (14):

25 $$T_{{\text{r}}} = T_{{\text{H}}} e^{{\frac{{\gamma \left( {V_{{\text{H}}} } \right)}}{q}\left[ {1 - \left( {\frac{V}{{V_{{\text{H}}} }}} \right)^{q} } \right]}}$$

This equation is the same as (14) except the starting point is the shock state.

**Fig. 4:**
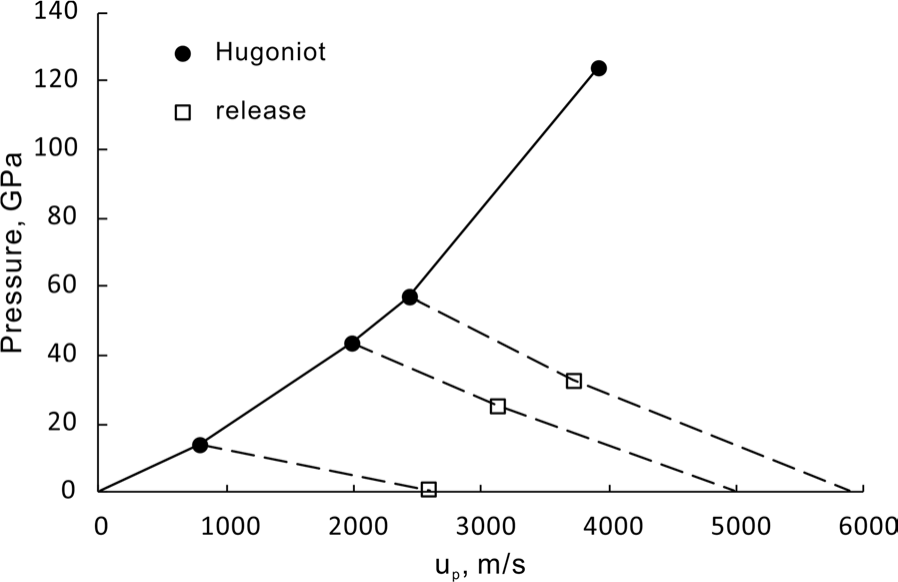
Measured Hugoniot and release state of the Bruderheim L6 chondrite in particle velocity–pressure space, after Anderson and Ahrens 1998

**Fig. 5:**
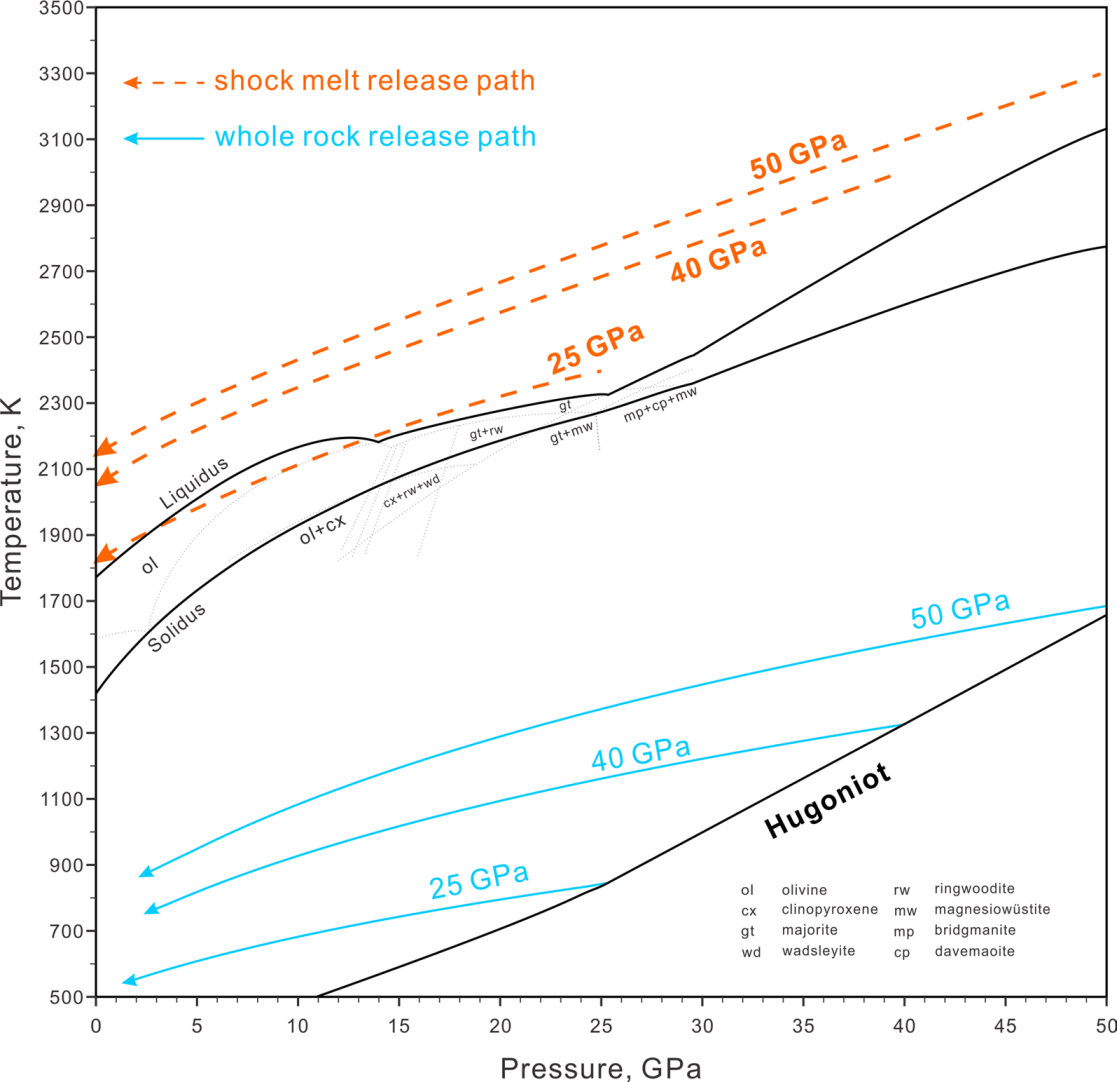
The adiabatic release paths of L chondrite bulk rock (cyan) and shock melt (orange) at 25, 40 and 50 GPa superimposed on the phase diagram of Agee et al. (1995). The *P–T* Hugoniot of L chondrite is the same as Figs. 2 and 3. The calculation parameters are in Tables 1 and 2

**Fig. 6:**
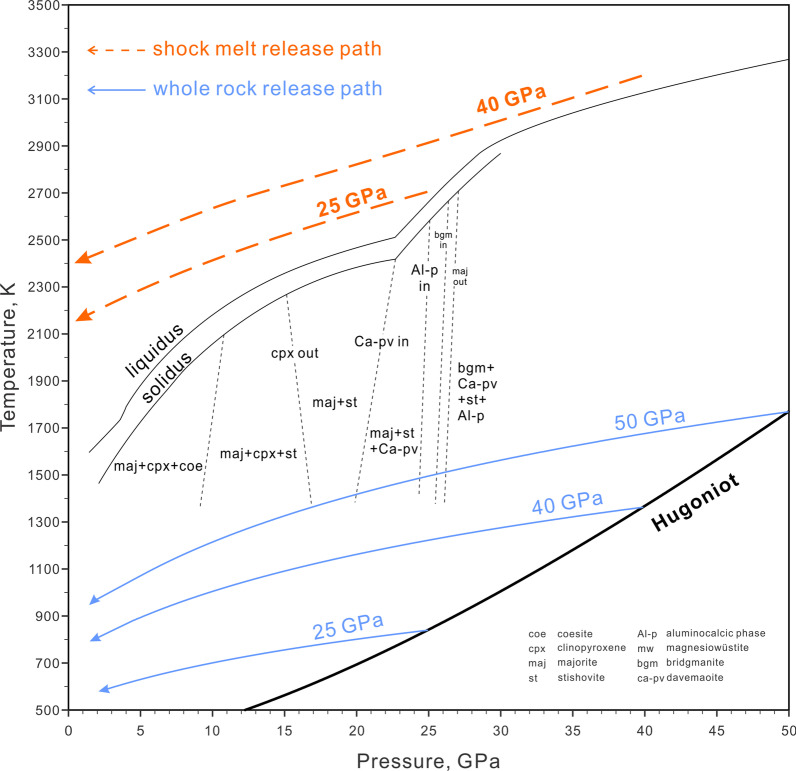
The adiabatic release path of bulk basalt (light purple) and basaltic melt (orange) superimposed on a phase diagram based on MORB from Fei and Bertka (1999) and Hirose et al. (1999). The P–T Hugoniot is the same as Fig. 3. Parameters used for the calculations are listed in Table 1 and 2

Anderson and Ahrens (1998) obtained three points on the release state of the Bruderheim L chondrite (Fig. 4). The data show that the sample has a higher particle velocity on the release path than that on the reflected Hugoniot. This indicates that the release volume is greater than the volume on the Hugoniot, based on the Riemann equation of release (Eq. 24). The interpretation can be that melting occurs more extensively in the sample than expected even at below 50 GPa (Fig. 3). In this scenario, for the unmelted portion, the final release volume should only be slightly greater than the initial condensed volume. With the limited data, it is difficult to build a complete release path. Instead, we use Eq. (25) to construct a *V*–*T* path of release for solid and melt. At the temperature of 500–800 K for the solid material, the expansion of major chondritic minerals is only on the order of 10^–3^ (Suzuki 1975) and we can use the initial volume as reference for release volume without making a noticeable error in the calculation of post-shock temperatures. The *P*–*T* release path is constructed by comparing the release path to the Hugoniot using the Mie-Grüneisen equation:

26 $$P_{{\text{r}}} \left( V \right) = P_{{\text{H}}} \left( V \right) + \frac{{\left( {T_{{\text{r}}} - T_{{\text{H}}} } \right)C_{{\text{V}}} \gamma }}{V}$$

where H and r denotes the points on the Hugoniot and release isentrope at volume V. The *P*–*T* release path of an L chondrite and a basalt from 25, 40 and 50 GPa are shown in Fig. 5 and 6. The slope of release is gentle at the beginning and becomes steeper with decreasing pressure. The complete temperature drop upon release is 300 (for 25 GPa)–900 (for 50 GPa) Kelvin in the bulk rock, making the post-shock temperature 500–900 K.

In meteorites, many shock features, particularly the high-pressure minerals, are associated with local shock melt (e.g. Sharp and DeCarli 2006; Tomioka and Miyahara 2017 and references therein). The *P*–*T* release path of shock melt is essential for understanding the formation of shock features. However, local melting represents a deviation from the bulk Hugoniot that can be very large at modest shock pressures. In this case, it is not possible to constrain the temperature and density of shock melt vein at < 50 GPa using the bulk Hugoniot of the rock. Qualitatively, we assume that the shock melt reaches pressure equilibrium with the bulk rock and has a shock temperature slightly higher than the liquidus for the sample (Figs. 5 and 6). In practice, the temperature of shock melt can be either significantly above the liquidus, resulting in further melting of adjacent host rock (Sharp et al. 2015), or it can be even slightly below the liquidus, because of metastable melting of low-pressure phases at high pressure.

To calculate the *P*–*T* release path of shock melt, we use the Grüneisen parameter and density of melt (Table 2) for Eq. (25). We refer to the density model of peridotite melt (Asimow 2018) to represent the chondritic melt. The data for basaltic melt are taken from ab initio simulations (Bajgain et al. 2015) as it cannot be easily measured by shock experiments. The Grüneisen parameter of a liquid is very different from that of a solid and commonly has a negative exponential factor *q* (Eq. 12; Asimow 2012). Parameters used for the calculations are listed in Table 2. The release paths from 25, 40, and 50 GPa (Fig. 5 and 6) show a moderate temperature drop and are mostly more gentle than the low-pressure liquidus of chondrite and basalt, indicating no crystallization upon adiabatic decompression in most cases. Although the chondrite path of 25 GPa crosses the liquidus between 4 and 13 GPa, crystallization is likely to be limited during adiabatic decompression because the release of latent heat of crystallization. For the basalt, the release path sits well above the liquidus through the pressure range (Fig. 6).

**Table 2:** Parameters and temperatures of shock melt

*P*, GPa	Chondrite	Basalt	Source
	25–50	25–40	
*γ*_o_	0.81	0.356	1
*q*	− 1.41	− 1.63	1
*ρ*, g/cm^3^	3.7–4.1	3.9–4.2	2, 3
*ρ*_o_, g/cm^3^	2.8	2.5	2, 3
*T*_shock_, K	2400–3300	2700–3200	
*T*_ps_, K	1815–2143	2140–2394	

*γ*_o_ and *q*: zero-pressure Grüneisen parameter and the exponent factor. *ρ* and *ρ*_o_: density at high and zero pressure. The shock temperature *T*_shock_ of melt is assumed to be slightly above the liquidus. Post-shock temperature *T*_ps_ is calculated using the listed parameters

^1^Asimow (2012); ^2^ Asimow (2015); ^3^ Bajgain et al. (2015)

## Temperature effects in shocked meteorites

**Fig. 7:**
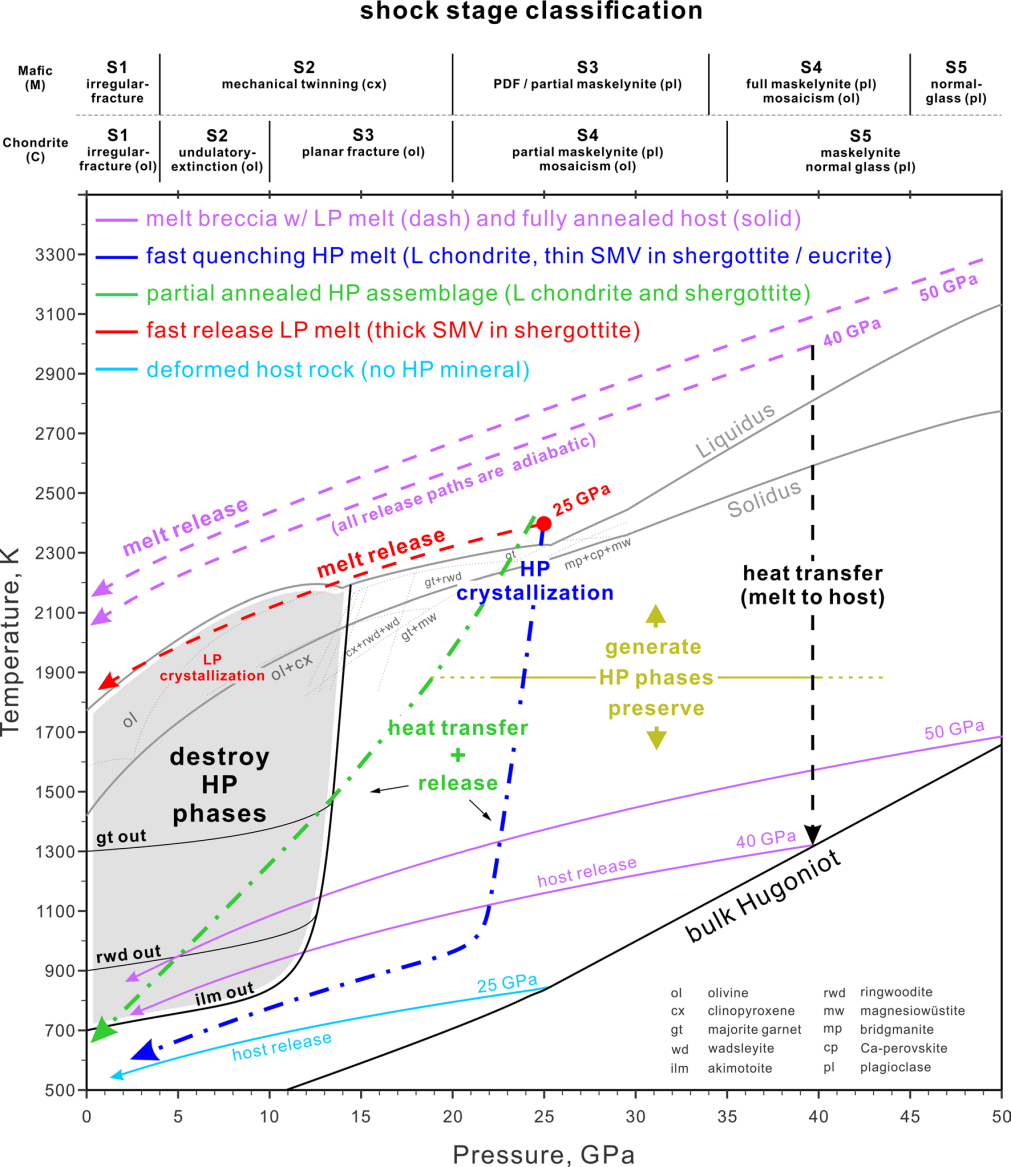
The cooling paths of typical moderately to highly shocked meteorites. The adiabatic release paths (cyan, red and purple lines) are from Fig. 5. HP and LP melt refer to shock melt that crystallizes before and after pressure release, respectively. The vertical line represents pure heat transfer from shock melt veins (SMV) to solid groundmass without synchronous pressure drop by decompression. The dark yellow line shows the approximated temperature of triggering rapid HP transformation. The grey area represents the conditions for destruction of akimotoite (ilm), ringwoodite (rwd) and garnet (gt) by amorphization or back transformation. The blue and green lines represent combinations of decompression and synchronous heat-transfer, where blue corresponds to rapid thermal quench with crystallization at high pressure and green corresponds to a greater contribution for decompression, resulting in crystallization at slightly lower pressure and partial annealing in the grey area. The shown *P–T* paths are technically for chondritic/ultramafic rocks but the concepts also work for mafic rocks. The top x-axis shows shock stage classification with deformation features of chondrite (C) and mafic meteorite (M) such as basaltic shergottite (Stöffler et al. 1991, 2018)

Our calculations of shock and release temperatures allow for the interpretation of shock effects in meteorites in terms of pressure–temperature–time (*P*–*T*–*t*) histories. The shocked meteorites that we will discuss include HP-mineral bearing (previously classified as S6 based on Stöffler et al. 1991) samples, partially annealed “S6” samples and melt breccias (some also represent S6) that have abundant shock melt but lack HP minerals. The *P–T* conditions of HP-mineral formation as well as *P–T* paths of quench and annealing are illustrated in Fig. 7. This discussion will walk through the representative samples, their corresponding paths in Fig. 7 and how their pressures deviate from the actual shock stage S6.

### HP-mineral formation constraints on P–T

**Fig. 8:**
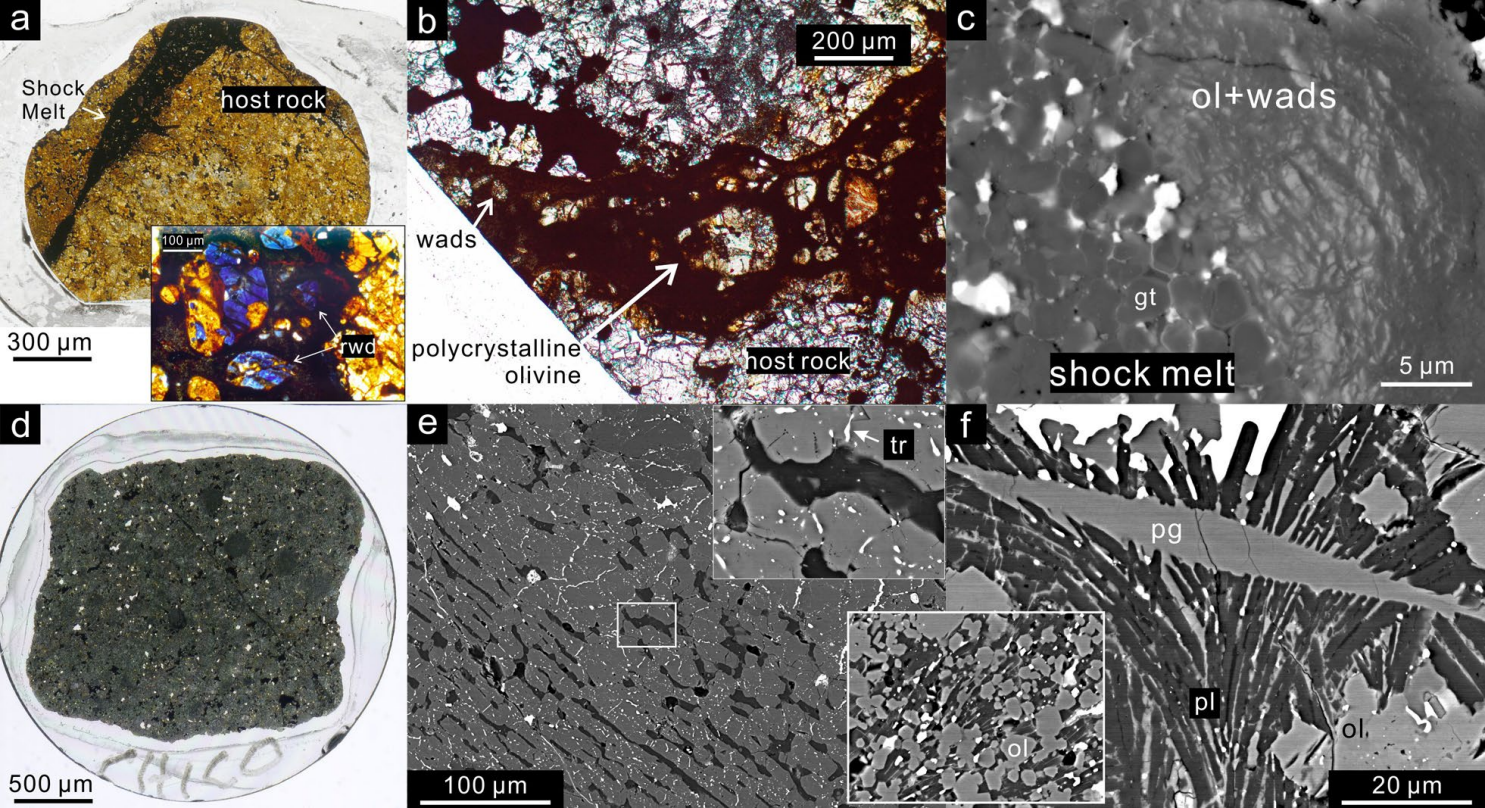
Variable shock features in L chondrites previously classified as shock stage S5-6. **a** RC 106 (L6) thin section with a thick shock melt vein. The insert is a plane-polarized light (PPL) micrograph of the shock melt vein containing blue ringwoodite (rwd). **b** PPL micrograph of a shock melt vein in Mbale (L5-6). The clasts in the thin part of vein are wadsleyite (wads) aggregates and the thicker vein contains polycrystalline olivine. **c** backscattered electron (BSE) image of a polycrystalline olivine (ol) aggregate with trace wadsleyite in contact with majorite garnet (gt) in quenched Mbale shock melt. **d** Shock-darkened impact melt breccia Chico (L6). **e** BSE image of a barred olivine chondrule in melt breccia NWA 091 (L6). The troilite (tr) veinlets are disseminated in the olivine. **f** BSE image of recrystallized sodic pigeonite (pg)-plagioclase (pl)-olivine (ol) aggregate in Chico. The insert is another recrystallized olivine-plagioclase aggregate. Figures modified after Sharp et al. (2015), Hu (2016) and Hu and Sharp (2017)

The majority of high-pressure minerals in shocked meteorites are formed by melt-crystallization and reconstructive phase transformations exclusively in melt regions and are mostly used to identify the S6 shock stage, based on Stöffler et al. 1991. The HP-mineral assemblage in most of these samples consists of polymorphs of common silicate, oxide and phosphate minerals (Sharp and DeCarli, 2006; Gillet and El Goresy 2013; Tomioka and Miyahara 2017 and references therein). Olivine clasts, entrained in the shock melt, are commonly transformed to ringwoodite or in some cases wadsleyite (Fig. 8a-b). The transformation of enstatite to its HP polymorphs majorite, akimotoite and bridgmanite are relatively less commonly reported. Shock melt in S6 L chondrites commonly crystallizes an assemblage that contains majoritic garnet solid solutions along with ringwoodite and/or magnesiowüstite (Fig. 8c) and in some cases bridgmanite or akimotoite. Such HP-mineral assemblages provide three major constraints on the pressure–temperature conditions of shock, as outlined in the following paragraphs.

First, the crystallization assemblages in these nominally highly shocked (S6) samples are consistent with crystallization at approximately 20–25 GPa, as indicated by static high-pressure experiments on chondritic compositions and the resulting liquidus phase-relations (Agee et al. 1995; Asahara et al. 2004). If a high-pressure crystallization assemblage in the sample is constant across the melt veins and melt pockets, it can be inferred that the pressure did not drop significantly during crystallization and that the crystallization pressure is the equilibration shock pressure (blue curve in Fig. 7; Sharp and De Carli 2006; Miyahara et al. 2011a; Hu and Sharp 2017). A constant or nearly constant crystallization pressure implies that the sample was in the isobaric zone of the impact where the separation between the shock wave and release wave results in a period of nearly constant equilibrium shock pressure. This isobaric zone is in contrast to areas shocked by an attenuated wave, which experiences an immediate decay in shock pressure instead of an initially constant pressure (Asimow 2015; Fritz et al. 2017). If the melt matrix includes mostly wadsleyite instead of ringwoodite, such as in LL chondrite NWA 757 (Hu and Sharp 2017), the crystallization pressure is inferred to be lower than 20 GPa. Even though metastable melting and crystallization may occur, the crystallization pressure can still be constrained from the liquidus and sub-solidus phase-assemblages in the melt matrix (Hu and Sharp 2017). In contrast to the long pulse of up to seconds in L chondrites, the relatively short pulse recorded in martian meteorites results in rapid pressure release before complete crystallization of the shock melt, leading to low-pressure crystallization assemblages and even vesiculated melt pockets (e.g. Walton et al. 2014; red melt release path in Fig. 7). It is worth noting that the short pulse does not necessarily imply small source craters on Mars. Shock dwell-time models by Bowling et al. (2020) indicate that dwell times of 10–100 ms are consistent with craters of 14–104 km diameter for a typical impact velocity of 13.1 km/s. Therefore, the 3–60 km potential meteorite-launching young (< 10 Ma) craters on Mars (Lagain et al. 2021) are consistent with relatively short pulse durations of tens of milliseconds. Nevertheless, the martian basalt NWA 8159 has a constant crystallization assemblage in a relatively thick melt vein that suggests a ~ 100 ms shock pulse (Sharp et al. 2019) and a somewhat larger source crater.

The large temperature difference between the super liquidus shock melt and relatively cool host rock drives the rapid high-pressure quench of the shock melt by heat conduction. The heat flux is proportional to temperature gradient, based on Fourier’s law. The heat transfer is also enhanced by turbulence of the shock melt that can transport hotter material to the interface with the colder bulk host (Adcock et al. 2017). The shock melt starts with a super liquidus temperature at ~ 2400 K (red dot in Fig. 7). The bulk shock-temperature of the host rock is only 500–700 K (cyan path) at 20–25 GPa, corresponding to a temperature difference of up to 1900 K. This large temperature gradient between the shock melt and the surrounding host rock results in the rapid transfer of heat into the surrounding host rock (blue *P–T* path in Fig. 7) and rapid thermal quench of the shock melt (Langenhorst and Poirier 2000; Xie et al. 2006b). If the shock pulse is longer than the time required to quench the melt, the melt will crystallize a high-pressure assemblage (Sharp and De Carli 2006). If such a long shock pulse occurs for samples within the isobaric shock zone, there would be no significant decompression during thermal quench and the resulting isobaric crystallization (blue path) would result in a constant crystallization assemblage throughout the quenched melt. This is apparently the scenario for many highly shocked L chondrites that have constant or nearly constant crystallization assemblages, implying that the *P–T*–*t* path and crystallization was dominated by thermal quench at high pressure and nearly isobaric (e.g. Chen et al. 1996; Ohtani et al. 2004; Sharp et al. 2015).

Second, the chondrite fragments entrained in the shock melt are heated rapidly to enable reconstructive transformation to HP polymorphs during the shock pulse (Fig. 8a). Although the duration of the shock pulse varies depending on the size of the impacting body and the location of the sample relative to the impact, the pulse duration is thought to be on the order of 1 s for highly shocked L chondrites (Chen et al. 2004; Ohtani et al. 2004; Beck et al. 2005; Sharp et al. 2015). Transformation of olivine on such a time scale requires a temperature in excess of 1600 K (Xie and Sharp 2007), illustrated by the dark yellow line and upward arrow in Fig. 7. The transformation of enstatite clasts to majorite, akimotoite or bridgmanite requires even higher temperatures because of the slow kinetics of enstatite transformations (Hogrefe et al. 1994; Lockridge 2015). The upward dark yellow arrow in Fig. 7 illustrates the rapid heating of an entrained mineral fragment to near solidus temperatures. Only material within the shock melt or immediately adjacent to the melt reach sufficiently high temperatures for reconstructive transformation during the shock pulse (Fig. 8a–c). The much rarer displacive transformation, such as zircon to reidite, can occur without extensive melting although higher local temperature would still greatly enhance the reaction (Leroux et al. 1999; Xing et al. 2020).

Thermal history is the key difference between HP mineral regions (previously S6) and the bulk host rock. Observable transformation of olivine or pyroxene cannot occur in the host-rock away from the shock melt because the bulk-shock temperature is only 600–800 K at 25 GPa (cyan path in Fig. 7). The relatively low temperature of the host rock limits the shock metamorphic effects to mosaicism (S4–S5), planar deformation features (S4–S5) and maskelynite (S5) outside the shock melt (Stöffler et al. 1991). In the recently revised scheme (Stöffler et al. 2018), these shock features are re-classified as S3-S5 (Fig. 7). This is consistent with shock classification of 2280 samples by Bischoff et al. (2019), who found that 70% of their 53 chondrite samples that contain ringwoodite are classified as shock stage up to S4 in regions distant from the shock melt. Stöffler et al. (1991) proposed that S6 shock features may result from local pressure–temperature excursions and therefore higher shock pressure than the bulk rock. Although significant pressure heterogeneities are expected during compression, pressure variations “ring down” to the equilibrium shock pressure in a very short time relative to the duration of the shock pulse and the formation of HP minerals (Sharp and De Carli 2006). Hydrocode simulations of mesoscale shock effects in chondrites confirm that local pressure excursions occur around high-impedance components (Moreau et al. 2017, 2018) and that shock pressures equilibrate in 10–20 ns. Although local temperature excursions are required to generate S6 features, the HP minerals form after the pressure heterogeneities have equilibrated.

Third, the HP minerals must cool below a critical temperature before pressure release in order to survive. At ambient pressure, ringwoodite and wadsleyite break down at temperatures above 900 K and back-transform to olivine within seconds at *T* > 1200 K (Hu and Sharp 2017). The critical temperature for retrograde back transformation depends on the stability of the mineral and the kinetics of its back-transformation reaction. The critical temperature for garnet is ~ 1300 K, which makes garnet more resistant to back transformation than the olivine polymorphs (Thiéblot et al. 1998), and therefore a robust indicator of high pressure in partially annealed samples. Pyroxene’s other two polymorphs, akimotoite and bridgmanite, break down at significantly lower temperatures of 700 and 400 K, respectively (Ashida et al. 1988; Durben and Wolf, 1992) and bridgmanite is generally transformed to glass during or after decompression (Sharp et al, 1997). The grey area in Fig. 7 represents a critical pressure–temperature region of annealing for akimotoite through garnet. In highly shocked samples that retain HP minerals, heat transfer from the melt zones to the relatively cool host rock is the major mechanism for quenching shock melt and for cooling HP minerals sufficiently to survive under reduced pressure conditions. Therefore, the *P–T*–*t* paths for quench of HP minerals must be steep (high d*T/*d*P*) in order to cool below the *P–T* conditions of back transformation (blue path in Fig. 7).

### Partially annealed samples

The back-transformation of HP minerals in some classic S6 samples implies shallow *P–T* paths (lower d*T/*d*P*) and insufficient cooling during pressure release. For example, EETA 79001 (shergottite) and Mbale (L chondrite) are highly shocked samples that contain fine-grained olivine aggregates, with remnants of ringwoodite or wadsleyite that are surrounded by radiating fractures from post-shock volume expansion (Walton 2013; Hu and Sharp 2017). These textures provide evidence of back-transformation of ringwoodite and wadsleyite and provide a mechanism for the origin of polycrystalline olivine aggregates. Remnants of high-pressure phases in the shock melt indicate that melt started at high pressure, but followed shallower *P–T* paths (green path in Fig. 7) that crossed through conditions of ringwoodite back transformation. The clasts in the shock melt were heated to temperatures sufficiently high to enable rapid transformation of olivine to its HP polymorphs (Fig. 8b, c) but did not cool below the critical temperature for back transformation before low-pressure conditions were reached. In these cases, rapid decompression occurred while heat was being transferred from the shock melt to the surrounding host rock. In Mbale (L5-6), thinner shock veins contain wadsleyite while thick veins contain back transformed olivine (Fig. 8 b and c), indicating that the slower-cooling thick vein had a shallower *P–T* path than the thinner vein (Hu and Sharp 2017).

The *P–T*–*t* path followed by any particular part of the sample will depend on the abundance of shock melt and the duration of the shock pulse. Graphically, the corresponding *P–T* paths (blue and green paths in Fig. 7) will have variable d*T*/d*P* slopes, depending on the amount of shock melt that is cooling during the shock pulse and release. For example a small melt vein or a melt vein edge would cool quickly by conduction and turbulence to the cooler host rock and would follow a relatively steep *P–T* path (high pressure portion of blue and green lines in Fig. 7). Similarly, a larger volume of melt contains higher internal energy that takes longer to dissipate and would therefore follow a relatively shallow *P–T* path (green line in Fig. 7) that may traverse the back-transformation regime of low-*P* and high-*T* (grey region). If the sample quenched in the isobaric shock zone, the paths would start with steep isobaric cooling, followed by shallowing of the *P–T*–*t* path. The extent of the isobaric cooling trend is inversely related to the amount of melt being cooled by conduction, such that the smallest volume of melt would produce a most stable isobaric segment for quenching before decompression. This is the case for many shocked L chondrites. In samples experiencing a short isobaric shock pulse, the *P–T*–*t* would start with a steep slope and quickly turn to a shallower slope (blue and green lines in Fig. 7). Outside of the isobaric zone, all *P–T*–*t* paths would be non-vertical decompression paths with the slope determined by the competing effects of decompression and thermal conduction to the cooler host rock. In shergottites, such as EETA 79,001, the shock pulse is inferred to be short and anisobaric, resulting in a *P–T* path through the back-transformation regime (Walton 2013). The back-transformed Mbale L chondrite, which crystallized a relatively small volume of melt at 14 – 18 GPa, is unlikely to have a lengthened cooling time caused by a high bulk-shock temperature (Hu and Sharp 2017). We therefore infer that Mbale experienced a shorter shock pulse than most HP-mineral bearing L chondrites (Hu and Sharp 2017). Furthermore, more pervasive melting and higher shock and post-shock temperature, as in impact melt breccias, would lead to more extensive back transformation and post-shock annealing.

Larger shock-melt volumes produce shallow *P–T*–*t* paths that are dominated by decompression. The red line in Fig. 7 illustrates the adiabatic cooling path for a shock melt at 25 GPa that decompresses without dissipation of heat to the host rock. This is not the case for HP-mineral-bearing “S6” L chondrites, which have small amounts of shock melt and steep *P–T*–*t* paths dominated by heat transfer. However, for small high-velocity impacts on planets, the shock pulse could be much shorter, resulting in decompression dominating the *P–T*–*t* quench path (Beck et al. 2005). Several shergottites have melt crystallization assemblages with olivine and pyroxene or glass coexisting with HP products of olivine transformation at ~ 25 GPa (Miyahara et al. 2011b, 2016; Walton 2013; Walton et al. 2014). In Tissint (olivine-phyric shergottite) for instance, only the micron thin veins crystallize and preserve stishovite and ringwoodite whereas the mm thick veins consist of olivine and clinopyroxene (Walton et al. 2014). The low-pressure assemblage of their quenched shock melts indicates that the corresponding *P–T*–*t* paths of the melt are closer to the adiabatic release path of melt (red line in Fig. 7).

### Impact melt breccias

Impact melt breccias have abundant shock features and evidence of high temperature, but they lack direct evidence of high pressure phase transitions. Sample such as NWA 091 and Chico (Bogard et al. 1995; Yolcubal et al. 1997; Hu 2016), have shock features that include recrystallization of olivine, blackening of silicates by disseminated metal-sulfide, feldspathic normal glass and pervasive impact melt (Fig. 8d–f). These samples were classified as highly shocked S6 samples by Stöffler et al. (1991), although they lack typical S6 shock veins and HP minerals. Bischoff et al (2019) classified Chico as an L6 breccia that contains fragments of melt breccia and we have studied a portion of melt breccia. Unlike a hand sample with pressure equilibrium in nanoseconds, the 105 kg Chico can have portions experiencing different levels of shock. The direct evidence for strong shock in these samples includes common recrystallization of rock-forming minerals and disseminated troilite that indicates mobile sulfide melt throughout the sample (Hu 2016). The troilite in these samples occurs as strings of tiny inclusions in olivine and pyroxene rather than veins (Fig. 8e). We interpret this texture to indicate that the silicate minerals were fractured during shock and the fractures were filled by sulfide melt. During subsequent annealing, the fractures healed, leaving strings of sulfide inclusions where fractures had been (Rubin 1992).

The severity of shock recorded in impact melt breccias is not well defined, but pervasive melting implies significantly higher shock pressures than those of S6-bearing chondrites. Moreau et al. (2017, 2018) used mesoscale hydrocode simulations to investigate the shock behavior of olivine-troilite-iron mixtures that resembles ordinary chondrites. The simulations at bulk shock pressures of 40–50 GPa result in considerable post-shock melting of troilite and the onset of local olivine melting. Moreau et al. (2017) inferred that > 35 GPa is the shock pressure range for pervasive silicate blackening. This is consistent with our interpretation that extensive darkening requires higher pressures than those of HP-mineral bearing “S6” samples (< 30 GPa). In blackened chondrite samples such as Chico, the sulfide particles and veinlets are much finer-grained than the common crack-filling sulfide in other chondrites and the sulfide particles occur in strings without co-existing fractures. We suggest that some chondrite melt breccias, including Chico and NWA 091, underwent high degrees of recrystallization and annealing at high post-shock temperatures (Hu 2016).

Shock pressures of 40–50 GPa would result in abundant shock melt, shallow *P–T*–*t* paths and post-shock temperatures too high for preservation of HP minerals (Fig. 7). Chico contains polycrystalline olivine-pyroxene-plagioclase aggregates (Fig. 8e, f) that appear to result from a combination of back-transformation, recrystallization and low-pressure melt crystallization (solid and dashed purple lines in Fig. 7). During a shock pulse to 40–50 GPa, the olivine single crystals in the shock melt would transform to polycrystalline aggregates of wadsleyite, ringwoodite or bridgmanite plus oxide (Ito and Takahashi 1989). During pressure release from 40 to 50 GPa, these HP minerals would follow a shallow *P–T* path between that of melt and the bulk rock (purple lines in Fig. 7). Based on observations in partially annealed samples, this would result in post-shock temperatures high enough to back-transform ringwoodite to olivine and cause recrystallization of back-transformed aggregates (Fig. 8f). Shock pressure greater than ~ 50 GPa would result in large melt fractions, generally shallower *P*–*T* decompression paths (Hu and Sharp 2017), and post-shock annealing out of high-shock indicators (grey area in Fig. 7). It is worth noting that, although the melt breccia may still contain thin shock veins that experienced relatively fast cooling with steeper *P*–*T* curves, the bulk post-shock temperature of the sample sits in the back-transformation regime and destroys the HP minerals.

Shock to 50 GPa results in a bulk release *P–T*–*t* path and a post shock temperature that is too hot for the preservation of most HP minerals. The bulk release path of 50 GPa (solid purple line in Fig. 7), which ends in the grey back-transformation area in Fig. 7, represents the lowest thermal history possible during and right after shock in such a sample. This illustrates that even the coldest parts of such a sample would experience back transformation of HP minerals and recrystallization of low-pressure mineral aggregates (Fig. 8e and f). The release paths for pure melt (dashed purple lines in Fig. 7) are above the liquidus and would result in crystallization at low pressure after pressure release. In natural melt-breccia samples, the release paths would be between these two end-members, with shock melt following a very shallow *P–T* path close to the liquidus. In this scenario, shock-melt crystallization may occur at elevated pressures, but at pressures generally too low to produce typical HP minerals and any HP minerals formed by transformation would not survive the high post-shock temperatures.

## Shock pressure estimation by ***P***–***T***–***t*** paths

Realistic *P–T*–*t* paths of shock metamorphism in chondrites are important for the interpretation of heterogeneous shock effects and conditions that are well off the bulk Hugoniot, particularly for the temperatures. The formation of shock melt veins results in local regions that are far hotter than that predicted by the bulk-rock Hugoniot for a given shock pressure. Formation of these local melt zones likely involves transient local pressure excursions, as described by Stöffler et al. (1991) but these transient pressure variations ring down to the equilibrated shock pressure at least 10^6^ times faster than the crystallization of even micron-sized shock melt (Langenhorst and Poirier 2000). More importantly, these local excursions, caused by pore collapse and shear during compression, result in temperature heterogeneities that are far above the temperature of the bulk-rock Hugoniot (Sharp and DeCarli 2006; Moreau et al. 2018). These temperature heterogeneities not only produce local melting and HP minerals; they also enhance thermally activated deformational processes. For example, the pressure for solid-state amorphization of plagioclase is strongly temperature dependent (Daniel et al. 1997; Tomioka et al. 2010; Kubo et al. 2010; Sharp et al. 2019) and the pressure at which PDFs and mosaicism occur decreases with increasing temperature (Huffman et al. 1993; Bauer 1979; Bowden 2002). Moreover, ignoring temperature heterogeneities would lead to difficulties explaining the significant discrepancy between co-existing shock features in many samples, such as sharp extinction of plagioclase in S4-S6 chondrites (Hu and Sharp 2017; Fritz et al. 2017). The thermal dependence of shock metamorphic effects explains why they are strongest (S6) in and adjacent to shock veins, but decrease to S5 and S4 with increasing distance from shock veins in some samples (Bischoff et al. 2019).

Given that some parts of a shocked sample deviate from the bulk Hugoniot conditions and are therefore “non-equilibrium” shock features (Stöffler et al.^1991^), they are still useful for estimating shock pressure because they are hot enough to drive thermally activated mineral reactions. For a typical HP-mineral bearing sample, the shock pressure needs to be roughly in the range as the stability field of the observed HP minerals, i.e. 15–30 GPa. In principle, HP minerals can form metastably at lower pressure than equilibrium under high strain (Melosh 1989) or by a metastable reaction (Walton et al. 2014; Kubo et al. 2015). The shock melt must experience sufficiently high pressure to crystallize HP minerals and contain enough heat to transform entrained clasts to HP phases. In the 15–30 GPa pressure range, the bulk shock temperature (Hugoniot temperature) is low enough for the bulk sample to act as a very large heat sink that drives rapid quench of the shock melt before and during decompression. These local “non-equilibrium” S6 shock features are essential for creating and preserving mineralogical evidence of high pressure.

If shock pressure were significantly higher than the 15–30 GPa indicated by HP mineral stability, the mineralogical evidence would not be well preserved (Fig. 7). For a shock pressure between 25 and 35 GPa, we would expect bridgmanite and magnesiowüstite to be the dominant HP minerals to crystallize from the shock melt and to form from the transformation of entrained mineral fragments. However, bridgmanite has the lowest preservation temperature of all of the HP minerals and readily decomposes at ambient pressure and temperatures in excess of ~ 400 K (Durben and Wolf 1992). This preservation temperature is lower than the post-shock release temperatures from 25 GPa (Fig. 6). Although bridgmanite can form in shocked meteorites, the shock conditions where bridgmanite should dominate the mineralogy cannot preserve bridgmanite after pressure release. This explains why crystalline bridgmanite is so rare in shocked chondrites, having only been found as a trace component in Tenham (L6; Tomioka and Fujino 1997; Tschauner et al. 2014). Instead, glassy grains with bridgmanite stoichiometry have been reported to coexist with akimotoite and ringwoodite in the quenched shock melt of Acfer 040 and Tenham (L6; Sharp et al. 1997; Xie et al. 2006b). The texture, composition and amorphous state provide strong evidence that these glassy grains were bridgmanite that crystallized from the shock melt and decomposed upon pressure release. Also, evidence for the transformation of olivine to bridgmanite (now pyroxene glass) plus magnesiowüstite has been reported from Martian meteorites, including Tissint (Walton et al, 2014; Miyahara et al. 2016; Ma et al. 2016) and Dar al Gani 735 (Miyahara et al. 2011b). If highly shocked meteorites were shocked to pressures above 25 GPa, we would expect to find abundant evidence for amorphous or transformed bridgmanite in S6 samples. The rarity of this evidence suggests that most shocked meteorites with S6 shock effects did not experience equilibrated shock pressures in excess of about 25 GPa. For a shock pressure of 40–50 GPa, even the bulk release temperature would be high enough to destroy all HP minerals (solid purple line in Fig. 7). In samples shocked to these pressures, the primary evidence for shock metamorphism would be extensive melting, darkening and recrystallization, as in the impact melt breccias.

### S6 shock-pressure inconsistencies and the use of HP minerals for evaluating shock stage classification

Shock deformation features in the unmelted portion of the meteorites have been the gold standard for shock classification and pressure estimation since Stöffler et al (1991) published their shock classification. Shock recovery experiments are an important technique for calibrating the corresponding shock pressures of these deformational features (e.g. Milton and DeCarli 1963; Stöffler 1972; Kieffer et al. 1976; Bauer 1979; Jeanloz 1980; Ostertag 1983; Stöffler and Langenhorst 1994) and they provide the basis for most of the Stöffler et al (1991) pressure calibration. However, S6 features, particularly HP minerals, are not well calibrated by shock recovery experiments. S6 shock effects, which are closely associated with shock melting, are interpreted by Stöffler et al (1991) to represent shock pressures from 50 to 90 GPa, with ringwoodite requiring 80–90 GPa to form. However, pinning of the pressure scale to 80–90 GPa for ringwoodite is not based on the formation of ringwoodite in shock recovery experiments, but rather on the lack of such shock synthesis. Without well calibrated pressures for S6 shock effects, this interpretation of S6 shock conditions, based on high-pressure minerals, is not well supported.

Chen et al. (1996) used TEM to characterize garnet-bearing melt-vein assemblages in Sixiangkou (L6) and the entrained ringwoodite and majorite that formed from olivine and enstatite. In that paper, the majorite-pyrope garnet plus magnesiowüstite assemblage was interpreted to represent melt-vein crystallization at 20–24 GPa and between 2050 and 2300 °C. This shock-pressure estimate was far lower than the 80–90 GPa estimate of ringwoodite formation of Stöffler et al. (1991) and started a heated discussion over the meaning of S6 shock effects and the use of HP mineral stability to constrain shock pressure. Here we use our modeling of shock temperatures and discussions of *P–T*–*t* cooling paths to provide insight into the importance of HP minerals and the S6 classification of shocked meteorites.

The central issue in the discussion of S6 features is the interpretation of shock-melt crystallization pressure versus the equilibrium shock pressure. Stöffler et al. (1991) state that S6 features represent local pressure–temperature excursions, within and near shock melt, but they are less clear about how to interpret those excursions in the context of equilibrium shock pressure. S6 features, especially blue ringwoodite aggregates in shock veins, have become an easily observed indicator of S6 shock stage. More recently, Stöffler et al. (2018) stated that HP minerals in shocked meteorites are duration-dependent and represent an extended pressure release regime that should be ignored in the interpretation of equilibrium shock pressures and they removed the use of HP minerals from S6 criteria and from most of the revised classification schemes. In particular, HP minerals are excluded from ultramafic and mafic samples (e.g. shergottites, eucrites etc.) and only mentioned as a minor possibility for S5-6 in chondrites (> 35 GPa), in contrast to an exclusively S6-defining feature in Stöffler et al. (1991). Although HP minerals do not support the pressure calibration of S6 in Stöffler et al (1991) or S5-6 in Stöffler et al. (2018), they do provide valuable constraints on shock pressure. The durations of non-equilibrium pressure excursions are on the order of 10–20 ns, which is too short for significant HP-mineral crystallization or transformation at extreme pressure. Instead, the high temperatures generated are much more important in creating HP-mineral signatures because they persist long after pressure equilibration. These high temperature zones, which are far off the bulk Hugoniot, are nearly the only parts of the samples hot-enough to record a mineralogical signature of shock pressure.

Because shock deformation features, such as fracturing and mosaicism, can occur in a wide range of shock pressures (e.g. Bauer 1979), HP mineral assemblages provide complementary constraints on shock pressure, temperature and duration that should not be ignored. The timing of shock-melt crystallization relative to pressure release is critical for interpreting pressure constraints from HP minerals. Many highly shocked L6 chondrites have nearly constant shock-vein crystallization assemblages that suggest a narrow range of crystallization pressure (e.g. < 15–25 GPa; Chen et al. 1996; Sharp and DeCarli 2006; Chen and Xie 2008; Miyahara et al. 2011a; Hu and Sharp 2017). This implies that the shock-veins in most highly shocked L chondrites represent crystallization at equilibrium shock pressures between 15 and 25 GPa. Crystallization during decompression cannot reconcile the difference between very high pressures for S6 features of Stöffler et al. (1991, 2018) and the 15–25 GPa crystallization pressures recorded in L chondrites. The *P–T*–*t* paths for S6 shock veins are driven by a combination of decompression and heat transfer to the surrounding bulk rock (blue and green lines in Fig. 7). It is highly unlikely that shock-melts formed at pressures in excess of 50 GPa and temperatures over 3000 K would all follow *P–T*–*t* paths that consistently cross the liquidus between 15 and 25 GPa regardless of the size and cooling rate of the melt zone. Such a large drop in crystallization pressures would result in a large range of crystallization assemblages in a given sample and between samples. Moreover, a quench path from 50 GPa that crosses the liquidus at 15–25 GPa would result in *P–T–t* conditions and post-shock temperatures that would destroy all HP minerals (solid purple lines in Fig. 7). The narrow pressure ranges recorded in many S6 L chondrites are consistent with shock in the isobaric zone (Fritz et al. 2017) of the large impact that broke up the L-chondrite parent body.

A similar scenario of HP minerals associated with shock melt occurs in the ultramafic–mafic achondrites, such as shergottites and eucrites, but these meteorites do not all record a narrow range of crystallization pressures. Martian meteorites commonly contain both high- and low-pressure features that represent crystallization and quenching through a range of release paths and shock outside of the isobaric zone (Walton et al. 2014; Fritz et al. 2017). This complication does not jeopardize the shock-pressure estimation from HP minerals but rather provides more information for constructing possible *P*–*T–t* paths for various parts of a given sample (green and red paths in Fig. 7). A HP mineral assemblage in the shergottite Zagami was first reported by Langenhorst and Poirier (2000) and majorite plus ringwoodite/wadsleyite assemblages, similar to those in shocked chondrites, were first reported in SNCs by Malavergne et al. (2001). Since then, many HP minerals have been recognized in mafic/ultramafic achondrites, indicating shock pressures of 15–30 GPa (Tomioka and Miyahara 2017 and references therein). Even the post-stishovite silica polymorph seifertite (Sharp et al. 1997; El Goresy et al. 2008; Miyahara et al. 2013), which has a stability field of > 100 GPa (Grocholski et al. 2013), can form metastably from cristobalite within the 15–30 GPa pressure range (Kubo et al. 2015). This pressure range is in good agreement with the moderate deformational features, such as partial to full diaplectic glasses and weak mosaicism in mafic minerals, found in Martian meteorites and eucrites (e.g. Langenhorst and Poirier 2000; Pang et al. 2016; Sharp et al. 2019). As mentioned above, shocked achondrites generally record shorter HP pulses, which makes the back-transformation and decompression sequence clearer in these samples (e.g. Walton 2013; Sharp et al. 2019; Hu et al. 2020). These occurrences are inconsistent with HP minerals originating from local/transient pressure excursions (Fritz and Greshake 2009). The samples truly shocked to ~ 40 GPa and above, would have high bulk temperature, pervasive melting and no HP minerals (e.g. ALHA 77,005; Walton and Herd 2007), similar to chondritic melt breccias. The claim that all the meteoritical HP minerals formed during pressure release from extreme pressures is not supported by observed assemblages. Therefore HP minerals can and should be used as shock stage indicator of moderate pressure in ultramafic–mafic achondrites.

The reconciliation of S6 conditions in shocked meteorites comes from the heterogeneous nature of shock effects and shock stage in these samples. Bischoff et al. (2019) reported shock classification results from 2280 shocked ordinary chondrites and specifically addressed the issue of shock-stage heterogeneity and S6 classification. They found that among 52 L chondrites and one LL chondrite with ringwoodite, 23 had more crystalline than amorphous plagioclase and an additional 16 had more than 25% crystalline plagioclase. This indicates that more than 70% of the ringwoodite-bearing chondrites belong to the shock stage S4 (Bischoff et al, 2019). They also stress the importance of sampling when classifying heterogeneous samples and the need to look at more than one small thin section in classifying shock stage. For example, Tenham, the iconic S6 chondrite which retains crystalline bridgmanite (Tschauner et al. 2014), has melt-vein free material that is classified as S4 (Bischoff et al. 2019). These results indicate that the S6 classification based on HP minerals is not a valid part of a progressive series of shock effects, but rather the result of local hot zones in samples shocked to predominantly S4 conditions. The 15–30 GPa (majorite predominant) shock pressures inferred from the crystallization of shock melt in previous S6 samples is well within the range of S4 conditions originally proposed by Stöffler et al. (1991). The presence of HP minerals, including the easily recognized presence of ringwoodite transformed from olivine, are not an indication of very high shock pressures, but rather an indication of moderate S4 shock pressures. Therefore, HP minerals in shock veins should not be ignored in shock classification because they provide evidence of moderate shock pressure and constraints on the *P–T–t* history of the shock melt through quench and decompression.

## Conclusions

High-pressure minerals are important signatures of shock in meteorites, but they are not necessarily an indication of very high shock pressures. Their production and preservation requires complex but well-constrained *P–T–t* paths from very high temperatures during shock to low temperature after pressure release. Calculations of shock and release temperatures provide the context for constraining these complex *P–T–t* paths. Observed high-pressure minerals in shocked meteorites correspond to shock pressures of 15–30 GPa. The corresponding HP liquidus for chondrites is approximately 2300 K, which is hot enough to enable the transformation of low-pressure minerals, in association with shock melt, to their high-pressure polymorphs. At these pressures, the bulk shock temperature is low enough for the host sample to act as a heat sink to rapidly cool shock-melt veins and pockets, resulting in rapid quench (high d*T*/d*P*) of these hot zones before and during decompression. The bulk meteorite is also sufficiently cool so that only deformational shock features occur outside the melt veins. In contrast, if a meteorite sample experiences even slightly hotter background temperature and/or shorter shock pulse, the quench of shock melt during decompression would have a gentle d*T*/d*P* slope which can leave the newly-formed HP assemblage too hot to survive at reduced pressure. The shock veins in samples with abundant melt, such as melt breccias, generally crystallize low-pressure assemblages and anneal out many shock deformation effects. At 40–50 GPa, the bulk shock temperature is too high for HP minerals to cool sufficiently to survive at low pressure. Samples shocked to such high pressures will only retain high-temperature features, such as extensive melting, darkening and recrystallization. The presence of ringwoodite in many chondritic samples, which was commonly considered as evidence of shock stage S6, is not an indication of very-high shock pressure, but rather an indication of moderate shock pressures consistent with shock stage S4.

## Data Availability

All data generated or analyzed during this study are included in this published article.

## Abbreviations

HP: High pressure
SMV: Shock melt vein
PDF: Planar deformation features
MORB: Mid-ocean ridge basalt
SNC: Shergottites, nakhlites, chassignites
